# Supplementary material for: Field size as a predictor of “excellence.” The selection of subject fields in Germany’s Excellence Initiative
Source: PLoS One. 2025 Mar 11;20(3):e0300828. doi: 10.1371/journal.pone.0300828 (PMC11896035; doi:10.1371/journal.pone.0300828)
Supplement: S1 Appendix — (DOCX) [file pone.0300828.s001.docx]

**Appendix 1: List of technical universities (alphabetical)**

| Brandenburgische Technische Universität Cottbus |
| --- |
| Gottfried Wilhelm Leibniz Universität Hannover |
| Karlsruher Institut für Technologie |
| Rheinisch-Westfälische Technische Hochschule Aachen |
| Technische Universität Bergakademie Freiberg |
| Technische Universität Berlin |
| Technische Universität Braunschweig |
| Technische Universität Chemnitz |
| Technische Universität Clausthal |
| Technische Universität Darmstadt |
| Technische Universität Dortmund |
| Technische Universität Dresden |
| Technische Universität Hamburg-Harburg |
| Technische Universität Ilmenau |
| Technische Universität Kaiserslautern |
| Technische Universität München |
| Universität Stuttgart |
