# Supplementary material for: Field size as a predictor of “excellence.” The selection of subject fields in Germany’s Excellence Initiative
Source: PLoS One. 2025 Mar 11;20(3):e0300828. doi: 10.1371/journal.pone.0300828 (PMC11896035; doi:10.1371/journal.pone.0300828)
Supplement: S2 Appendix — (DOCX) [file pone.0300828.s002.docx]

**Appendix 2: List of non-technical universities (alphabetical)**

| Albert-Ludwigs-Universität Freiburg |
| --- |
| Bergische Universität Wuppertal |
| Carl von Ossietzky Universität Oldenburg |
| Christian-Albrechts-Universität zu Kiel |
| Eberhard Karls Universität Tübingen |
| Ernst-Moritz-Arndt-Universität Greifswald |
| Europa-Universität Viadrina Frankfurt (Oder) |
| Freie Universität Berlin |
| Friedrich-Alexander-Universität Erlangen-Nürnberg |
| Friedrich-Schiller-Universität Jena |
| Georg-August-Universität Göttingen |
| Goethe-Universität Frankfurt am Main |
| Heinrich-Heine-Universität Düsseldorf |
| Humboldt-Universität zu Berlin |
| Johannes Gutenberg-Universität Mainz |
| Julius-Maximilians-Universität Würzburg |
| Justus-Liebig-Universität Gießen |
| Ludwig-Maximilians-Universität München |
| Otto-Friedrich-Universität Bamberg |
| Otto-von-Guericke-Universität Magdeburg |
| Philipps-Universität Marburg |
| Rheinische Friedrich-Wilhelms-Universität Bonn |
| Ruprecht-Karls-Universität Heidelberg |
| Universität Augsburg |
| Universität Bayreuth |
| Universität Bielefeld |
| Universität Bochum |
| Universität Bremen |
| Universität des Saarlandes |
| Universität Duisburg-Essen |
| Universität Erfurt |
| Universität Halle |
| Universität Hamburg |
| Universität Hohenheim |
| Universität Kassel |
| Universität Koblenz-Landau |
| Universität Konstanz |
| Universität Leipzig |
| Universität Lüneburg |
| Universität Mannheim |
| Universität Osnabrück |
| Universität Paderborn |
| Universität Passau |
| Universität Potsdam |
| Universität Regensburg |
| Universität Rostock |
| Universität Siegen |
| Universität Trier |
| Universität Ulm |
| Universität zu Köln |
| Westfälische Wilhelms-Universität Münster |
