# Supplementary material for: Field size as a predictor of “excellence.” The selection of subject fields in Germany’s Excellence Initiative
Source: PLoS One. 2025 Mar 11;20(3):e0300828. doi: 10.1371/journal.pone.0300828 (PMC11896035; doi:10.1371/journal.pone.0300828)
Supplement: S3 Appendix — (DOCX) [file pone.0300828.s003.docx]

**Appendix 3: Data from the Federal Statistical Office (StBA)**

Outliers and missing data in the personnel, financial and student variables were identified and missing values were imputed wherever possible in an otherwise steady trend. Outliers that differed by several orders of magnitude from the preceding and following years were recoded as missing values.

The staff statistics from the Federal Statistical Office identify five non-overlapping categories for professorial staff: “temporary professors,” “C4/W3 professors,” “C3/W2 professors,” “C2 professors,” and “junior professors.” These categories constitute the variable “professors” in our data set.

The financial statistics from the Federal Statistical Office distinguish between two categories of external funding: “public grants” and “grants from other areas,” which together constitute the variable “revenue from grants.” They have been adjusted for inflation (base year: 2005).

The student statistics from the Federal Statistical Office differentiate among registered students according to their study objective (target degree). We have used the available categories to produce the variable “total students,” which comprises students working toward Bachelor’s and Master’s degrees as well as students aiming for a teaching degree.
