# Supplementary material for: Field size as a predictor of “excellence.” The selection of subject fields in Germany’s Excellence Initiative
Source: PLoS One. 2025 Mar 11;20(3):e0300828. doi: 10.1371/journal.pone.0300828 (PMC11896035; doi:10.1371/journal.pone.0300828)
Supplement: S4 Appendix — (DOCX) [file pone.0300828.s004.docx]

# Appendix 4: Aggregation scheme of StBA examination groups

|  | Aggregated group | Examination groups (standard font), final examinations (*italic*) |
| --- | --- | --- |
| X | University degree (without teacher training examinations) | *Master degree; licentiate; ecclesiastical examination; state examination/1^st^ state examination; state examination (single-phase education); faculty examination; diploma (U); diploma (U-GH); diploma (U) - interpreter; diploma (U) - translator; certified translator; diploma (U) - teacher; final examination without academic degree* |
| X | Bachelor degree (without teacher training examinations) | *Multi-field bachelor without teacher training option; multi-field bachelor with teacher training option; bachelor (U) - teacher; bachelor at universities; bachelor at art colleges; bachelor at universities of applied sciences* |
| X | Master degree (without teacher training examinations) | *Multi-field master; master (U) – teacher; master at universities (final examination required); master at art colleges (final examination required); master at universities of applied sciences (final examination required)* |
| X | Teacher training examinations | Teacher training, bachelor and master examinations (TT/BA/MA) at primary and lower secondary schools/primary level; teacher training, bachelor and master examinations (TT/BA/MA) at intermediate level (secondary level I/primary schools/primary level); teacher training, bachelor and master examinations (TT/BA/MA) at secondary schools/technical secondary level I; teacher training, bachelor and master examinations (TT/BA/MA) comprehensive examination for secondary level II/secondary level I; teacher training, bachelor and master examinations (TT/BA/MA) at grammar school/secondary level II, general schools; teacher training, bachelor and master examinations (TT/BA/MA) at special schools; teacher training, bachelor and master examinations (TT/BA/MA) at vocational schools/secondary level II, vocational schools; TT bachelor; TT master; other TT according to school types/school levels |
|  | Doctorates | Doctorates |
|  | Artistic degree | Artistic degree (not including *bachelor degree at art colleges* or *master degree at art colleges (final examination required)*) |
|  | University of Applied Sciences degree | University of Applied Sciences degree (not including *bachelor degree at universities of applied sciences* or *master degree at universities of applied sciences (final examination required)*) |
|  | Other academic qualification | Other academic qualification |

Note: X indicates that we have included the aggregated group (StBA) in our analysis.

Source: StBA. Bildung und Kultur. Studierende an Hochschulen. Fachserie 11, Reihe 4.1. Wiesbaden: Statistisches Bundesamt; 1992-2016.
