# Supplementary material for: Field size as a predictor of “excellence.” The selection of subject fields in Germany’s Excellence Initiative
Source: PLoS One. 2025 Mar 11;20(3):e0300828. doi: 10.1371/journal.pone.0300828 (PMC11896035; doi:10.1371/journal.pone.0300828)
Supplement: S5 Appendix — (DOCX) [file pone.0300828.s005.docx]

# Appendix 5: Concordance table of StBA with Archambault classification

| **Field classification of the StBA** | **Archambault subfield** |
| --- | --- |
| Agricultural Sciences, Food and Beverages Technology | Agronomy & Agriculture |
| Agricultural Sciences, Food and Beverages Technology | Food Science |
| Agricultural Sciences, Food and Beverages Technology | Horticulture |
| Agricultural Sciences, Food and Beverages Technology | Dairy & Animal Science |
| Biology | Toxicology |
| Biology | Bioinformatics |
| Biology | Biotechnology |
| Biology | Anthropology |
| Biology | Biochemistry & Molecular Biology |
| Biology | Biophysics |
| Biology | Developmental Biology |
| Biology | Genetics & Heredity |
| Biology | Microbiology |
| Biology | Ecology |
| Biology | Entomology |
| Biology | Evolutionary Biology |
| Biology | Marine Biology & Hydrobiology |
| Biology | Ornithology |
| Biology | Plant Biology & Botany |
| Biology | Zoology |
| Chemistry | Analytical Chemistry |
| Chemistry | General Chemistry |
| Chemistry | Inorganic & Nuclear Chemistry |
| Chemistry | Medicinal & Biomolecular Chemistry |
| Chemistry | Organic Chemistry |
| Chemistry | Physical Chemistry |
| Chemistry | Polymer |
| Chemistry | Nanoscience & Nanotechnology |
| Electrical Engineering | Optoelectronics & Photonics |
| Electrical Engineering | Electrical & Electronic Engineering |
| Electrical Engineering | Networking & Telecommunications |
| Nutrition and Home Economics | Dairy & Animal Science |
| Nutrition and Home Economics | Nutrition & Dietetics |
| Forestry, Timber Management | Forestry |
| Geosciences (excluding Geography) | Geological & Geomatics Engineering |
| Geosciences (excluding Geography) | Geochemistry & Geophysics |
| Geosciences (excluding Geography) | Geology |
| Geosciences (excluding Geography) | Meteorology & Atmospheric Sciences |
| Geosciences (excluding Geography) | Oceanography |
| Geosciences (excluding Geography) | Palaeontology |
| Geosciences (excluding Geography) | Environmental Sciences |
| Mechanical Engineering/Process Engineering | Energy |
| Mechanical Engineering/Process Engineering | Biomedical Engineering |
| Mechanical Engineering/Process Engineering | Chemical Engineering |
| Mechanical Engineering/Process Engineering | Materials |
| Mechanical Engineering/Process Engineering | Environmental Engineering |
| Mechanical Engineering/Process Engineering | Industrial Engineering & Automation |
| Mechanical Engineering/Process Engineering | Mechanical Engineering & Transports |
| Mathematics | Applied Mathematics |
| Mathematics | General Mathematics |
| Mathematics | Numerical & Comp. Mathematics |
| Mathematics | Statistics & Probability |
| Physics, Astronomy | Acoustics |
| Physics, Astronomy | Applied Physics |
| Physics, Astronomy | Astronomy & Astrophysics |
| Physics, Astronomy | Chemical Physics |
| Physics, Astronomy | Fluids & Plasmas |
| Physics, Astronomy | General Physics |
| Physics, Astronomy | Mathematical Physics |
| Physics, Astronomy | Nuclear & Particles Physics |
| Physics, Astronomy | Optics |
| Physics, Astronomy | Nanoscience & Nanotechnology |
| Psychology | Behav. Science & Comp. Psychology |
| Psychology | Clinical Psychology |
| Psychology | Developmental & Child Psychology |
| Psychology | Experimental Psychology |
| Psychology | Gene Psychology & Cognitive Sciences |
| Psychology | Human Factors |
| Psychology | Social Psychology |
| Economics | Operations Research |
| Economics | Accounting |
| Economics | Agricultural Economics & Policy |
| Economics | Business & Management |
| Economics | Development Studies |
| Economics | Econometrics |
| Economics | Economic Theory |
| Economics | Economics |
| Economics | Finance |
| Economics | Industrial Relations |
| Economics | Logistics & Transportation |
| Economics | Marketing |
| Economics | Sports, Leisure & Tourism |
