# Supplementary material for: Field size as a predictor of “excellence.” The selection of subject fields in Germany’s Excellence Initiative
Source: PLoS One. 2025 Mar 11;20(3):e0300828. doi: 10.1371/journal.pone.0300828 (PMC11896035; doi:10.1371/journal.pone.0300828)
Supplement: S6 Appendix — (DOCX) [file pone.0300828.s006.docx]

# Appendix 6. Assignment of Excellence Initiative funding to subject fields

| **University** | **Funding line** | **Subject field** |
| --- | --- | --- |
| Albert-Ludwigs-Universität Freiburg | EC1 | Biology |
| Albert-Ludwigs-Universität Freiburg | EC2 | Biology |
| Albert-Ludwigs-Universität Freiburg | EC2 | Electrical Engineering |
| Albert-Ludwigs-Universität Freiburg | EC2 | Computer Science |
| Albert-Ludwigs-Universität Freiburg | GS1 | Biology |
| Albert-Ludwigs-Universität Freiburg | GS1 | Chemistry |
| Albert-Ludwigs-Universität Freiburg | GS1 | Medicine |
| Albert-Ludwigs-Universität Freiburg | GS2 | Biology |
| Albert-Ludwigs-Universität Freiburg | GS2 | Chemistry |
| Albert-Ludwigs-Universität Freiburg | GS2 | Medicine |
| Albert-Ludwigs-Universität Freiburg | IS1 |  |
| Carl von Ossietzky Universität Oldenburg | EC2 | Biology |
| Carl von Ossietzky Universität Oldenburg | EC2 | Medicine |
| Carl von Ossietzky Universität Oldenburg | EC2 | Physics |
| Christian-Albrechts-Universität zu Kiel | EC1 | Biology |
| Christian-Albrechts-Universität zu Kiel | EC1 | Geosciences |
| Christian-Albrechts-Universität zu Kiel | EC1 | Medicine |
| Christian-Albrechts-Universität zu Kiel | EC1 | Physics |
| Christian-Albrechts-Universität zu Kiel | EC1 | Economics |
| Christian-Albrechts-Universität zu Kiel | EC2 | Biology |
| Christian-Albrechts-Universität zu Kiel | EC2 | Geosciences |
| Christian-Albrechts-Universität zu Kiel | EC2 | Medicine |
| Christian-Albrechts-Universität zu Kiel | EC2 | Physics |
| Christian-Albrechts-Universität zu Kiel | EC2 | Economics |
| Christian-Albrechts-Universität zu Kiel | GS1 | Biology |
| Christian-Albrechts-Universität zu Kiel | GS1 | History |
| Christian-Albrechts-Universität zu Kiel | GS2 | Biology |
| Christian-Albrechts-Universität zu Kiel | GS2 | History |
| Eberhard Karls Universität Tübingen | EC1 | Medicine |
| Eberhard Karls Universität Tübingen | EC1 | Computer Science |
| Eberhard Karls Universität Tübingen | EC2 | Medicine |
| Eberhard Karls Universität Tübingen | EC2 | Computer Science |
| Eberhard Karls Universität Tübingen | GS2 | Biology |
| Eberhard Karls Universität Tübingen | GS2 | Medicine |
| Eberhard Karls Universität Tübingen | GS2 | Psychology |
| Eberhard Karls Universität Tübingen | IS2 |  |
| Freie Universität Berlin | EC1 | Classical Philology |
| Freie Universität Berlin | EC1 | History |
| Freie Universität Berlin | EC1 | Medicine |
| Freie Universität Berlin | EC1 | Psychology |
| Freie Universität Berlin | EC1 | Social Sciences |
| Freie Universität Berlin | EC2 | Classical Philology |
| Freie Universität Berlin | EC2 | History |
| Freie Universität Berlin | EC2 | Medicine |
| Freie Universität Berlin | GS1 | General and Comparative Literature Studies |
| Freie Universität Berlin | GS1 | English and American Language and Literature Studies |
| Freie Universität Berlin | GS1 | History |
| Freie Universität Berlin | GS1 | Other Language and Cultural Studies |
| Freie Universität Berlin | GS2 | General and Comparative Literature Studies |
| Freie Universität Berlin | GS2 | English and American Language and Literature Studies |
| Freie Universität Berlin | GS2 | Biology |
| Freie Universität Berlin | GS2 | Chemistry |
| Freie Universität Berlin | GS2 | History |
| Freie Universität Berlin | GS2 | Computer Science |
| Freie Universität Berlin | GS2 | Mathematics |
| Freie Universität Berlin | GS2 | Physics |
| Freie Universität Berlin | GS2 | Other Language and Cultural Studies |
| Freie Universität Berlin | IS1 |  |
| Freie Universität Berlin | IS2 |  |
| Friedrich-Alexander-Universität Erlangen-Nürnberg | EC1 | Mechanical Engineering |
| Friedrich-Alexander-Universität Erlangen-Nürnberg | EC2 | Mechanical Engineering |
| Friedrich-Alexander-Universität Erlangen-Nürnberg | GS1 | Biology |
| Friedrich-Alexander-Universität Erlangen-Nürnberg | GS1 | Chemistry |
| Friedrich-Alexander-Universität Erlangen-Nürnberg | GS1 | Physics |
| Friedrich-Alexander-Universität Erlangen-Nürnberg | GS2 | Biology |
| Friedrich-Alexander-Universität Erlangen-Nürnberg | GS2 | Chemistry |
| Friedrich-Alexander-Universität Erlangen-Nürnberg | GS2 | Physics |
| Friedrich-Schiller-Universität Jena | GS1 | Biology |
| Friedrich-Schiller-Universität Jena | GS2 | Biology |
| Georg-August-Universität Göttingen | EC1 | Biology |
| Georg-August-Universität Göttingen | EC1 | Chemistry |
| Georg-August-Universität Göttingen | EC1 | Physics |
| Georg-August-Universität Göttingen | EC2 | Biology |
| Georg-August-Universität Göttingen | EC2 | Chemistry |
| Georg-August-Universität Göttingen | EC2 | Physics |
| Georg-August-Universität Göttingen | GS1 | Biology |
| Georg-August-Universität Göttingen | GS1 | Chemistry |
| Georg-August-Universität Göttingen | GS2 | Biology |
| Georg-August-Universität Göttingen | GS2 | Chemistry |
| Georg-August-Universität Göttingen | IS1 |  |
| Goethe-Universität Frankfurt am Main | EC1 | Biology |
| Goethe-Universität Frankfurt am Main | EC1 | Chemistry |
| Goethe-Universität Frankfurt am Main | EC1 | History |
| Goethe-Universität Frankfurt am Main | EC1 | Medicine |
| Goethe-Universität Frankfurt am Main | EC1 | Philosophy |
| Goethe-Universität Frankfurt am Main | EC1 | Political Science |
| Goethe-Universität Frankfurt am Main | EC2 | Biology |
| Goethe-Universität Frankfurt am Main | EC2 | Chemistry |
| Goethe-Universität Frankfurt am Main | EC2 | History |
| Goethe-Universität Frankfurt am Main | EC2 | Medicine |
| Goethe-Universität Frankfurt am Main | EC2 | Philosophy |
| Goethe-Universität Frankfurt am Main | EC2 | Political Science |
| Gottfried Wilhelm Leibniz Universität Hannover | EC1 | Physics |
| Gottfried Wilhelm Leibniz Universität Hannover | EC2 | Biology |
| Gottfried Wilhelm Leibniz Universität Hannover | EC2 | Medicine |
| Gottfried Wilhelm Leibniz Universität Hannover | EC2 | Physics |
| Heinrich-Heine-Universität Düsseldorf | EC2 | Biology |
| Humboldt-Universität zu Berlin | EC1 | Classical Philology |
| Humboldt-Universität zu Berlin | EC1 | History |
| Humboldt-Universität zu Berlin | EC1 | Medicine |
| Humboldt-Universität zu Berlin | EC2 | Classical Philology |
| Humboldt-Universität zu Berlin | EC2 | History |
| Humboldt-Universität zu Berlin | EC2 | Medicine |
| Humboldt-Universität zu Berlin | GS1 | General and Comparative Literature Studies |
| Humboldt-Universität zu Berlin | GS1 | Biology |
| Humboldt-Universität zu Berlin | GS1 | Chemistry |
| Humboldt-Universität zu Berlin | GS1 | Philosophy |
| Humboldt-Universität zu Berlin | GS1 | Physics |
| Humboldt-Universität zu Berlin | GS1 | Political Science |
| Humboldt-Universität zu Berlin | GS1 | Psychology |
| Humboldt-Universität zu Berlin | GS1 | Social Sciences |
| Humboldt-Universität zu Berlin | GS2 | General and Comparative Literature Studies |
| Humboldt-Universität zu Berlin | GS2 | Biology |
| Humboldt-Universität zu Berlin | GS2 | Chemistry |
| Humboldt-Universität zu Berlin | GS2 | History |
| Humboldt-Universität zu Berlin | GS2 | Computer Science |
| Humboldt-Universität zu Berlin | GS2 | Mathematics |
| Humboldt-Universität zu Berlin | GS2 | Philosophy |
| Humboldt-Universität zu Berlin | GS2 | Physics |
| Humboldt-Universität zu Berlin | GS2 | Psychology |
| Humboldt-Universität zu Berlin | GS2 | Other Language and Cultural Studies |
| Humboldt-Universität zu Berlin | IS2 |  |
| Johannes Gutenberg-Universität Mainz | EC2 | Physics |
| Johannes Gutenberg-Universität Mainz | GS1 | Physics |
| Johannes Gutenberg-Universität Mainz | GS2 | Physics |
| Julius-Maximilians-Universität Würzburg | GS1 | Biology |
| Julius-Maximilians-Universität Würzburg | GS1 | Chemistry |
| Julius-Maximilians-Universität Würzburg | GS1 | Medicine |
| Julius-Maximilians-Universität Würzburg | GS2 | Biology |
| Julius-Maximilians-Universität Würzburg | GS2 | Chemistry |
| Julius-Maximilians-Universität Würzburg | GS2 | Medicine |
| Justus-Liebig-Universität Gießen | EC1 | Medicine |
| Justus-Liebig-Universität Gießen | EC2 | Medicine |
| Justus-Liebig-Universität Gießen | GS1 | English and American Language and Literature Studies |
| Justus-Liebig-Universität Gießen | GS1 | Language and Cultural Studies, general |
| Justus-Liebig-Universität Gießen | GS2 | Anglistik und Amerikanistik |
| Justus-Liebig-Universität Gießen | GS2 | English and American Language and Literature Studies |
| Karlsruher Institut für Technologie | EC1 | Physics |
| Karlsruher Institut für Technologie | GS1 | Physics |
| Karlsruher Institut für Technologie | GS2 | Physics |
| Karlsruher Institut für Technologie | IS1 |  |
| Ludwig-Maximilians-Universität München | EC1 | Biology |
| Ludwig-Maximilians-Universität München | EC1 | Chemistry |
| Ludwig-Maximilians-Universität München | EC1 | Physics |
| Ludwig-Maximilians-Universität München | EC2 | Biology |
| Ludwig-Maximilians-Universität München | EC2 | Chemistry |
| Ludwig-Maximilians-Universität München | EC2 | Medicine |
| Ludwig-Maximilians-Universität München | EC2 | Physics |
| Ludwig-Maximilians-Universität München | GS1 | Biology |
| Ludwig-Maximilians-Universität München | GS2 | Classical Philology |
| Ludwig-Maximilians-Universität München | GS2 | Biology |
| Ludwig-Maximilians-Universität München | GS2 | Medicine |
| Ludwig-Maximilians-Universität München | GS2 | Other Language and Cultural Studies |
| Ludwig-Maximilians-Universität München | IS1 |  |
| Ludwig-Maximilians-Universität München | IS2 |  |
| Otto-Friedrich-Universität Bamberg | GS2 | Political Science |
| Otto-Friedrich-Universität Bamberg | GS2 | Social Sciences |
| Rheinische Friedrich-Wilhelms-Universität Bonn | EC1 | Mathematics |
| Rheinische Friedrich-Wilhelms-Universität Bonn | EC2 | Biology |
| Rheinische Friedrich-Wilhelms-Universität Bonn | EC2 | Mathematics |
| Rheinische Friedrich-Wilhelms-Universität Bonn | GS1 | Physics |
| Rheinische Friedrich-Wilhelms-Universität Bonn | GS1 | Economics |
| Rheinische Friedrich-Wilhelms-Universität Bonn | GS2 | Physics |
| Rheinisch-Westfälische Technische Hochschule Aachen | EC1 | Electrical Engineering |
| Rheinisch-Westfälische Technische Hochschule Aachen | EC1 | Mechanical Engineering |
| Rheinisch-Westfälische Technische Hochschule Aachen | EC2 | Mechanical Engineering |
| Rheinisch-Westfälische Technische Hochschule Aachen | GS1 | Computer Science |
| Rheinisch-Westfälische Technische Hochschule Aachen | GS2 | Computer Science |
| Rheinisch-Westfälische Technische Hochschule Aachen | IS1 |  |
| Rheinisch-Westfälische Technische Hochschule Aachen | IS2 |  |
| Ruhr-Universität Bochum | EC2 | Chemistry |
| Ruprecht-Karls-Universität Heidelberg | EC1 | Biology |
| Ruprecht-Karls-Universität Heidelberg | EC1 | Chemistry |
| Ruprecht-Karls-Universität Heidelberg | EC1 | Cultural Studies in the narrow sense |
| Ruprecht-Karls-Universität Heidelberg | EC1 | Other Language and Cultural Studies |
| Ruprecht-Karls-Universität Heidelberg | EC2 | Medicine |
| Ruprecht-Karls-Universität Heidelberg | EC2 | Cultural Studies in the narrow sense |
| Ruprecht-Karls-Universität Heidelberg | EC2 | Other Language and Cultural Studies |
| Ruprecht-Karls-Universität Heidelberg | GS1 | Biology |
| Ruprecht-Karls-Universität Heidelberg | GS1 | Medicine |
| Ruprecht-Karls-Universität Heidelberg | GS1 | Mathematics |
| Ruprecht-Karls-Universität Heidelberg | GS1 | Physics |
| Ruprecht-Karls-Universität Heidelberg | GS2 | Biology |
| Ruprecht-Karls-Universität Heidelberg | GS2 | Medicine |
| Ruprecht-Karls-Universität Heidelberg | GS2 | Mathematics |
| Ruprecht-Karls-Universität Heidelberg | GS2 | Physics |
| Ruprecht-Karls-Universität Heidelberg | IS1 |  |
| Ruprecht-Karls-Universität Heidelberg | IS2 |  |
| Technische Universität Berlin | EC1 | Chemistry |
| Technische Universität Berlin | EC2 | Chemistry |
| Technische Universität Berlin | GS1 | Mathematics |
| Technische Universität Berlin | GS2 | Mathematics |
| Technische Universität Chemnitz | EC2 | Mechanical Engineering |
| Technische Universität Darmstadt | EC1 | Mechanical Engineering |
| Technische Universität Darmstadt | GS1 | Computer Science |
| Technische Universität Darmstadt | GS1 | Mathematics |
| Technische Universität Darmstadt | GS2 | Computer Science |
| Technische Universität Darmstadt | GS2 | Mechanical Engineering |
| Technische Universität Darmstadt | GS2 | Mathematics |
| Technische Universität Dresden | EC1 | Biology |
| Technische Universität Dresden | EC2 | Biology |
| Technische Universität Dresden | EC2 | Electrical Engineering |
| Technische Universität Dresden | EC2 | Computer Science |
| Technische Universität Dresden | GS1 | Biology |
| Technische Universität Dresden | GS1 | Computer Science |
| Technische Universität Dresden | GS1 | Mechanical Engineering |
| Technische Universität Dresden | GS2 | Biology |
| Technische Universität Dresden | GS2 | Computer Science |
| Technische Universität Dresden | GS2 | Mechanical Engineering |
| Technische Universität Dresden | IS2 |  |
| Technische Universität Kaiserslautern | GS2 | Physics |
| Technische Universität München | EC1 | Biology |
| Technische Universität München | EC1 | Chemistry |
| Technische Universität München | EC1 | Mechanical Engineering |
| Technische Universität München | EC1 | Physics |
| Technische Universität München | EC2 | Biology |
| Technische Universität München | EC2 | Chemistry |
| Technische Universität München | EC2 | Medicine |
| Technische Universität München | EC2 | Physics |
| Technische Universität München | IS1 |  |
| Technische Universität München | IS2 |  |
| Universität Augsburg | EC2 | Physics |
| Universität Bayreuth | GS1 | Other Language and Cultural Studies |
| Universität Bayreuth | GS2 | Other Language and Cultural Studies |
| Universität Bielefeld | EC1 | Biology |
| Universität Bielefeld | EC1 | Computer Science |
| Universität Bielefeld | EC1 | Sports Science |
| Universität Bielefeld | EC2 | Biology |
| Universität Bielefeld | EC2 | Computer Science |
| Universität Bielefeld | EC2 | Sports Science |
| Universität Bielefeld | GS1 | History |
| Universität Bielefeld | GS1 | Philosophy |
| Universität Bielefeld | GS1 | Social Sciences |
| Universität Bielefeld | GS2 | History |
| Universität Bielefeld | GS2 | Philosophy |
| Universität Bielefeld | GS2 | Social Sciences |
| Universität Bremen | EC1 | Geosciences |
| Universität Bremen | EC2 | Geosciences |
| Universität Bremen | GS1 | Biology |
| Universität Bremen | GS1 | Chemistry |
| Universität Bremen | GS1 | Political Science |
| Universität Bremen | GS1 | Psychology |
| Universität Bremen | GS1 | Social Sciences |
| Universität Bremen | GS2 | Political Science |
| Universität Bremen | GS2 | Psychology |
| Universität Bremen | GS2 | Social Sciences |
| Universität Bremen | IS2 |  |
| Universität des Saarlandes | EC1 | Computer Science |
| Universität des Saarlandes | EC2 | Computer Science |
| Universität des Saarlandes | GS1 | Computer Science |
| Universität des Saarlandes | GS2 | Computer Science |
| Universität Hamburg | EC1 | Geosciences |
| Universität Hamburg | EC2 | Chemistry |
| Universität Hamburg | EC2 | Geosciences |
| Universität Hamburg | EC2 | Physics |
| Universität Konstanz | EC1 | Philosophy |
| Universität Konstanz | EC1 | Political Science |
| Universität Konstanz | EC1 | Sociology |
| Universität Konstanz | EC2 | Philosophy |
| Universität Konstanz | EC2 | Political Science |
| Universität Konstanz | EC2 | Sociology |
| Universität Konstanz | GS1 | Biology |
| Universität Konstanz | GS1 | Chemistry |
| Universität Konstanz | GS2 | Biology |
| Universität Konstanz | GS2 | Chemistry |
| Universität Konstanz | GS2 | Political Science |
| Universität Konstanz | GS2 | Psychology |
| Universität Konstanz | GS2 | Economics |
| Universität Konstanz | IS1 |  |
| Universität Konstanz | IS2 |  |
| Universität Leipzig | GS1 | Physics |
| Universität Mannheim | GS1 | Social Sciences |
| Universität Mannheim | GS1 | Economics |
| Universität Mannheim | GS2 | Social Sciences |
| Universität Mannheim | GS2 | Economics |
| Universität Regensburg | GS2 | Other Language and Cultural Studies |
| Universität Stuttgart | GS1 | Computer Science |
| Universität Stuttgart | GS1 | Economics |
| Universität Stuttgart | GS2 | Computer Science |
| Universität Stuttgart | GS2 | Economics |
| Universität Ulm | GS1 | Medicine |
| Universität Ulm | GS2 | Medicine |
| Universität zu Köln | EC1 | Biology |
| Universität zu Köln | EC2 | Biology |
| Universität zu Köln | GS1 | Physics |
| Universität zu Köln | GS2 | Philosophy |
| Universität zu Köln | GS2 | Physics |
| Universität zu Köln | IS2 |  |
| Westfälische Wilhelms-Universität Münster | EC1 | Protestant Theology |
| Westfälische Wilhelms-Universität Münster | EC1 | Catholic Theology |
| Westfälische Wilhelms-Universität Münster | EC1 | Political Science |
| Westfälische Wilhelms-Universität Münster | EC2 | Biology |
| Westfälische Wilhelms-Universität Münster | EC2 | Chemistry |
| Westfälische Wilhelms-Universität Münster | EC2 | Protestant Theology |
| Westfälische Wilhelms-Universität Münster | EC2 | Medicine |
| Westfälische Wilhelms-Universität Münster | EC2 | Catholic Theology |
| Westfälische Wilhelms-Universität Münster | EC2 | Political Science |

Note: ECx are clusters of excellence; GSx are Graduate Research Schools; ISx are institutional strategies. The index x refers to the funding phase; x=1 is the first funding phase 2006-2011, x=2 the second phase 2012- 2017. The field “medicine” is for information only, because we did not process data on medical faculties due to the data problems mentioned in Section 3.
