# Supplementary material for: Field size as a predictor of “excellence.” The selection of subject fields in Germany’s Excellence Initiative
Source: PLoS One. 2025 Mar 11;20(3):e0300828. doi: 10.1371/journal.pone.0300828 (PMC11896035; doi:10.1371/journal.pone.0300828)
Supplement: S7 Appendix — (DOCX) [file pone.0300828.s007.docx]

# Appendix 7: Correlation matrices

Tab. 7a: Correlation matrix, first “initiative” phase (2006-2011), all universities

|  | Professors | Total grant funding | DFG grant funding | Students | Citations |
| --- | --- | --- | --- | --- | --- |
| Professors | 1.000 |  |  |  |  |
| Total grant funding | 0.578^***^ | 1.000 |  |  |  |
| DFG grant funding | 0.203^***^ | 0.215^***^ | 1.000 |  |  |
| Students | 0.688^***^ | 0.352^***^ | 0.201^***^ | 1.000 |  |
| Citations | 0.436^***^ | 0.438^***^ | 0.159^***^ | 0.106^***^ | 1.000 |
| Observations | 2,388 |  |  |  |  |

^*^ *p* < 0.05, ^**^ *p* < 0.01, ^***^ *p* < 0.001

Tab. 7b: Correlation matrix, second “initiative” phase (2012-2017), all universities

|  | Professors | Total grant funding | DFG grant funding | Students | Citations | Phase 1 |
| --- | --- | --- | --- | --- | --- | --- |
| Professors | 1.000 |  |  |  |  |  |
| Total grant funding | 0.607^***^ | 1.000 |  |  |  |  |
| DFG grant funding | 0.232^***^ | 0.214^***^ | 1.000 |  |  |  |
| Students | 0.730^***^ | 0.492^***^ | 0.195^***^ | 1.000 |  |  |
| Citations | 0.477^***^ | 0.477^***^ | 0.176^***^ | 0.159^***^ | 1.000 |  |
| Phase 1 | 0.329^***^ | 0.365^***^ | 0.179^***^ | 0.180^***^ | 0.430^***^ | 1.000 |
| Observations | 2,396 |  |  |  |  |  |

^*^ *p* < 0.05, ^**^ *p* < 0.01, ^***^ *p* < 0.001

Tab. 7c: Correlation matrix, first “initiative” phase (2006-2011), 12 subject fields with

good WoS coverage, all universities

|  | Professors | Total grant funding | DFG grant funding | Students | Citations |
| --- | --- | --- | --- | --- | --- |
| Professors | 1.000 |  |  |  |  |
| Total grant funding | 0.575^***^ | 1.000 |  |  |  |
| DFG grant funding | 0.334^***^ | 0.343^***^ | 1.000 |  |  |
| Students | 0.665^***^ | 0.356^***^ | 0.223^***^ | 1.000 |  |
| Citations | 0.403^***^ | 0.352^***^ | 0.343^***^ | 0.022 | 1.000 |
| Observations | 551 |  |  |  |  |

^*^ *p* < 0.05, ^**^ *p* < 0.01, ^***^ *p* < 0.001

Tab. 7d: Correlation matrix, second “initiative” phase (2012-2017), 12 subject fields with good WoS coverage, all universities

|  | Professors | Total grant funding | DFG grant funding | Students | Citations | Phase 1 |
| --- | --- | --- | --- | --- | --- | --- |
| Professors | 1.000 |  |  |  |  |  |
| Total grant funding | 0.590^***^ | 1.000 |  |  |  |  |
| DFG grant funding | 0.413^***^ | 0.355^***^ | 1.000 |  |  |  |
| Students | 0.737^***^ | 0.556^***^ | 0.252^***^ | 1.000 |  |  |
| Citations | 0.444^***^ | 0.392^***^ | 0.393^***^ | 0.065 | 1.000 |  |
| Phase 1 | 0.355^***^ | 0.400^***^ | 0.334^***^ | 0.178^***^ | 0.518^***^ | 1.000 |
| Observations | 550 |  |  |  |  |  |

^*^ *p* < 0.05, ^**^ *p* < 0.01, ^***^ *p* < 0.001
