# Supplementary material for: Field size as a predictor of “excellence.” The selection of subject fields in Germany’s Excellence Initiative
Source: PLoS One. 2025 Mar 11;20(3):e0300828. doi: 10.1371/journal.pone.0300828 (PMC11896035; doi:10.1371/journal.pone.0300828)
Supplement: S8 Appendix — (DOCX) [file pone.0300828.s008.docx]

# Appendix 8: Logistic regression analyses

Tab. 8a: Logistic regression, second “initiative” phase (2012-2017), NTUs

| DV=ExIn-funded | | | | | |
| --- | --- | --- | --- | --- | --- |
|  | Model 1 | Model 2 | Model 3 | Model 4 | Model 5 |
| Professors | 0.126599^***^ | 0.065732^***^ | 0.051643^***^ | 0.107650^***^ | 0.099668^**^ |
| Total grant funding |  | 0.298433^***^ | 0.296642^***^ | 0.233122^***^ | 0.214615^***^ |
| DFG grant funding |  |  | 0.036037^***^ | 0.035357^***^ | 0.025522^*^ |
| Students |  |  |  | -0.814180^**^ | -0.808057^*^ |
| Phase 1 |  |  |  |  | 5.587971^***^ |
| Intercept | -4.267726^***^ | -4.176585^***^ | -4.995859^***^ | -4.989408^***^ | -5.708097^***^ |
| Observations | 1872 | 1872 | 1872 | 1872 | 1872 |
| Pseudo-R2 | 0.200744 | 0.269628 | 0.299963 | 0.314449 | 0.642206 |

^*^ *p* < 0.05, ^**^ *p* < 0.01, ^***^ *p* < 0.001; ExIn = “excellence initiative”

Tab. 8b: Logistic regression, second “initiative” phase (2012-2017), TUs

| DV=ExIn-funded | | | | | |
| --- | --- | --- | --- | --- | --- |
|  | Model 1 | Model 2 | Model 3 | Model 4 | Model 5 |
| Professors | 0.145839^***^ | 0.129660^***^ | 0.126906^***^ | 0.170633^***^ | 0.210778^**^ |
| Total grant funding |  | 0.022429 | 0.021843 | 0.105880^*^ | 0.062580 |
| DFG grant funding |  |  | 0.006038 | 0.007909 | -0.059053 |
| Students |  |  |  | -1.332431^*^ | -1.755588 |
| Phase 1 |  |  |  |  | 7.673918^***^ |
| Intercept | -5.314181^***^ | -5.194264^***^ | -5.356676^***^ | -5.548694^***^ | -5.340610^***^ |
| Observations | 524 | 524 | 524 | 524 | 524 |
| Pseudo-R2 | 0.318055 | 0.321262 | 0.322242 | 0.359837 | 0.746805 |

^*^ *p* < 0.05, ^**^ *p* < 0.01, ^***^ *p* < 0.001; ExIn = “excellence initiative”

Tab. 8c: Logistic regression, first “initiative” phase (2006-2011), NTUs

| DV=ExIn-funded | | | | |
| --- | --- | --- | --- | --- |
|  | Model 1 | Model 2 | Model 3 | Model 4 |
| Professors | 0.102579^***^ | 0.055361^***^ | 0.047246^***^ | 0.060696^**^ |
| Total grant funding |  | 0.350526^***^ | 0.313880^***^ | 0.289041^***^ |
| DFG grant funding |  |  | 0.081932^***^ | 0.083187^***^ |
| Students |  |  |  | -0.175910 |
| Intercept | -4.145971^***^ | -4.154941^***^ | -5.352402^***^ | -5.367308^***^ |
| Observations | 1852 | 1852 | 1852 | 1852 |
| Pseudo-R2 | 0.132634 | 0.194125 | 0.236820 | 0.238270 |

^*^ *p* < 0.05, ^**^ *p* < 0.01, ^***^ *p* < 0.001; ExIn = “excellence initiative”

Tab. 8d: Logistic regression, first “initiative” phase (2006-2011), TUs

| DV=ExIn-funded | | | | |
| --- | --- | --- | --- | --- |
|  | Model 1 | Model 2 | Model 3 | Model 4 |
| Professors | 0.121399^***^ | 0.098810^***^ | 0.101597^***^ | 0.085094^**^ |
| Total grant funding |  | 0.051689 | 0.035792 | -0.007922 |
| DFG grant funding |  |  | 0.060482 | 0.054749 |
| Students |  |  |  | 0.869810^*^ |
| Intercept | -4.964031^***^ | -4.837065^***^ | -6.132478^***^ | -6.400819^***^ |
| Observations | 536 | 536 | 536 | 536 |
| Pseudo-R2 | 0.254066 | 0.264581 | 0.288912 | 0.319044 |

^*^ *p* < 0.05, ^**^ *p* < 0.01, ^***^ *p* < 0.001; ExIn = “excellence initiative”

Tab. 8e: Logistic regression, first “initiative” phase (2006-2011), natural sciences

| DV=ExIn-funded | | | | |
| --- | --- | --- | --- | --- |
|  | Model 1 | Model 2 | Model 3 | Model 4 |
| Professors | 0.155950^***^ | 0.082983^**^ | 0.077637^**^ | 0.075690^**^ |
| Total grant funding |  | 0.375925^***^ | 0.321290^***^ | 0.320879^***^ |
| DFG grant funding |  |  | 0.054876^**^ | 0.054596^*^ |
| Students |  |  |  | 0.071758 |
| Intercept | -4.543776^***^ | -4.534415^***^ | -5.285426^***^ | -5.295774^***^ |
| Observations | 514 | 514 | 514 | 514 |
| Pseudo-R2 | 0.235079 | 0.309631 | 0.328897 | 0.329021 |

^*^ *p* < 0.05, ^**^ *p* < 0.01, ^***^ *p* < 0.001; ExIn = “excellence initiative”

Tab. 8f: Logistic regression, second “initiative” phase (2012-2017), natural sciences

| DV=ExIn-funded | | | | | |
| --- | --- | --- | --- | --- | --- |
|  | Model 1 | Model 2 | Model 3 | Model 4 | Model 5 |
| Professors | 0.215851^***^ | 0.142938^***^ | 0.134247^***^ | 0.147732^***^ | 0.184459^***^ |
| Total grant funding |  | 0.221826^***^ | 0.213142^***^ | 0.210432^***^ | 0.088022 |
| DFG grant funding |  |  | 0.012736 | 0.013054 | -0.002499 |
| Students |  |  |  | -0.377013 | -0.299023 |
| Phase 1 |  |  |  |  | 4.729829^***^ |
| Intercept | -5.421236^***^ | -5.111836^***^ | -5.295268^***^ | -5.259958^***^ | -6.038445^***^ |
| Observations | 512 | 512 | 512 | 512 | 512 |
| Pseudo-R2 | 0.374617 | 0.415087 | 0.418316 | 0.420407 | 0.651497 |

^*^ *p* < 0.05, ^**^ *p* < 0.01, ^***^ *p* < 0.001; ExIn = “excellence initiative”

Tab. 8g: Logistic regression, first “initiative” phase (2006-2011), social sciences

| DV=ExIn-funded | | | | |
| --- | --- | --- | --- | --- |
|  | Model 1 | Model 2 | Model 3 | Model 4 |
| Professors | 0.052552^**^ | 0.034296 | 0.035050 | 0.090940 |
| Total grant funding |  | 0.430796^*^ | 0.363529 | 0.378447^*^ |
| DFG grant funding |  |  | 0.031375 | 0.036132 |
| Students |  |  |  | -0.537127 |
| Intercept | -3.826455^***^ | -3.933624^***^ | -4.389794^***^ | -4.493444^***^ |
| Observations | 431 | 431 | 431 | 431 |
| Pseudo-R2 | 0.043616 | 0.073950 | 0.082885 | 0.095758 |

^*^ *p* < 0.05, ^**^ *p* < 0.01, ^***^ *p* < 0.001; ExIn = “excellence initiative”

Tab. 8h: Logistic regression, second “initiative” phase (2012-2017), social sciences

| DV=ExIn-funded | | | | | | |
| --- | --- | --- | --- | --- | --- | --- |
|  | Model 1 | Model 2 | Model 3 | Model 4 | Model 5 |  |
| Professors | 0.049424^**^ | 0.018250 | 0.018541 | 0.129890^*^ | 0.053212 |  |
| Total grant funding |  | 0.383354 | 0.397720 | 0.543396^*^ | -0.102404 |  |
| DFG grant funding |  |  | -0.005972 | -0.008393 | -0.096110 |  |
| Students |  |  |  | -1.643999^*^ | -0.154471 |  |
| Phase 1 |  |  |  |  | 7.680742^***^ |  |
| Intercept | -3.913044^***^ | -3.906463^***^ | -3.781269^***^ | -3.676088^***^ | -3.878507^***^ |  |
| Observations | 435 | 435 | 435 | 435 | 435 |  |
| Pseudo-R2 | 0.045639 | 0.067744 | 0.068850 | 0.119069 | 0.617039 |  |

^*^ *p* < 0.05, ^**^ *p* < 0.01, ^***^ *p* < 0.001; ExIn = “excellence initiative”

Tab. 8i: Logistic regression, first “initiative” phase (2006-2011), humanities

| DV=ExIn-funded | | | | |
| --- | --- | --- | --- | --- |
|  | Model 1 | Model 2 | Model 3 | Model 4 |
| Professors | 0.096919^***^ | 0.066854^*^ | 0.059251 | 0.058412 |
| Total grant funding |  | 0.593877^*^ | 0.502999 | 0.501938 |
| DFG grant funding |  |  | 0.053168^*^ | 0.053203^*^ |
| Students |  |  |  | 0.013085 |
| Intercept | -4.229057^***^ | -4.277242^***^ | -5.082191^***^ | -5.083426^***^ |
| Observations | 892 | 892 | 892 | 892 |
| Pseudo-R2 | 0.055673 | 0.073600 | 0.095725 | 0.095733 |

^*^ *p* < 0.05, ^**^ *p* < 0.01, ^***^ *p* < 0.001; ExIn = “excellence initiative”

Tab. 8j: Logistic regression, second “initiative” phase (2012-2017), humanities

| DV=ExIn-funded | | | | | |
| --- | --- | --- | --- | --- | --- |
|  | Model 1 | Model 2 | Model 3 | Model 4 | Model 5 |
| Professors | 0.125874^***^ | 0.074985^*^ | 0.055988 | 0.074107^*^ | 0.065653 |
| Total grant funding |  | 0.578881^***^ | 0.521392^**^ | 0.512895^**^ | 0.350722 |
| DFG grant funding |  |  | 0.033264^**^ | 0.032935^**^ | 0.034625 |
| Students |  |  |  | -0.264743 | -1.074188 |
| Phase 1 |  |  |  |  | 7.708898^***^ |
| Intercept | -4.257597^***^ | -4.255603^***^ | -5.057668^***^ | -5.047063^***^ | -5.988166^***^ |
| Observations | 882 | 882 | 882 | 882 | 882 |
| Pseudo-R2 | 0.085978 | 0.121927 | 0.154723 | 0.156872 | 0.689805 |

^*^ *p* < 0.05, ^**^ *p* < 0.01, ^***^ *p* < 0.001; ExIn = “excellence initiative”

Tab. 8k: Logistic regression, first “initiative” phase (2006-2011), 12 subject fields with good WoS coverage, all universities

| DV=ExIn-funded | | | | | |
| --- | --- | --- | --- | --- | --- |
|  | Model 1 | Model 2 | Model 3 | Model 4 | Model 5 |
| Citations | 0.491121^***^ | 0.423491^***^ | 0.394527^***^ | 0.371862^***^ | 0.399359^***^ |
| DFG grant funding |  | 0.080448^***^ | 0.065535^***^ | 0.065521^***^ | 0.064095^**^ |
| Total grant funding |  |  | 0.082459^*^ | 0.058416 | 0.058690 |
| Professors |  |  |  | 0.024477 | 0.009057 |
| Students |  |  |  |  | 0.226920 |
| Intercept | -3.120918^***^ | -4.442549^***^ | -4.432054^***^ | -4.732205^***^ | -4.680096^***^ |
| Observations | 551 | 551 | 551 | 551 | 551 |
| Pseudo-R2 | 0.242012 | 0.293836 | 0.314653 | 0.318042 | 0.320264 |

^*^ *p* < 0.05, ^**^ *p* < 0.01, ^***^ *p* < 0.001; ExIn = “excellence initiative”

Tab. 8l: Logistic regression, second “initiative” phase (2012-2017), 12 subject fields with good WoS coverage, all universities

| DV=ExIn-funded | | | | | | |
| --- | --- | --- | --- | --- | --- | --- |
|  | Model 1 | Model 2 | Model 3 | Model 4 | Model 5 | Model 6 |
| Citations | 0.374516^***^ | 0.313515^***^ | 0.306052^***^ | 0.296377^***^ | 0.244042^***^ | 0.208071^***^ |
| Professors |  | 0.072413^***^ | 0.055470^**^ | 0.051237^**^ | 0.096048^***^ | 0.110432^**^ |
| Total grant funding |  |  | 0.041544 | 0.038220 | 0.069533^*^ | 0.032564 |
| DFG grant funding |  |  |  | 0.011302 | 0.010513 | -0.009075 |
| Students |  |  |  |  | -0.747593^*^ | -0.841682 |
| Phase 1 |  |  |  |  |  | 4.204282^***^ |
| Intercept | -3.141701^***^ | -4.282371^***^ | -4.169624^***^ | -4.354396^***^ | -4.516610^***^ | -4.739416^***^ |
| Observations | 550 | 550 | 550 | 550 | 550 | 550 |
| Pseudo-R2 | 0.303030 | 0.353190 | 0.361003 | 0.363783 | 0.376480 | 0.581031 |

^*^ *p* < 0.05, ^**^ *p* < 0.01, ^***^ *p* < 0.001; ExIn = “excellence initiative”
