# Supplementary material for: Field size as a predictor of “excellence.” The selection of subject fields in Germany’s Excellence Initiative
Source: PLoS One. 2025 Mar 11;20(3):e0300828. doi: 10.1371/journal.pone.0300828 (PMC11896035; doi:10.1371/journal.pone.0300828)
Supplement: S9 Appendix — (DOCX) [file pone.0300828.s009.docx]

# Appendix 9: Two-level logistic regression analyses

Tab. 9a: Two-level logistic regression, first “initiative” phase (2006-2011), all universities

| DV=ExIn-funded | | | | | | |
| --- | --- | --- | --- | --- | --- | --- |
|  | Model 1 | Model 2 | Model 3 | Model 4 | Model 5 | Model 6 |
| Professors | 0.114526^***^ | 0.084284^***^ | 0.081678^***^ | 0.096923^***^ | 0.096456^***^ | 0.096464^***^ |
| Total grant funding |  | 0.147767^***^ | 0.126985^***^ | 0.118584^**^ | 0.119499^**^ | 0.119510^**^ |
| DFG grant funding |  |  | 0.097759^***^ | 0.100229^***^ | 0.093319^**^ | 0.093028^**^ |
| Students |  |  |  | -0.264286 | -0.264427 | -0.264605 |
| Founded after 1945 (U) |  |  |  |  | -0.341890 | -0.345394 |
| East Germany (U) |  |  |  |  |  | -0.040420 |
| Intercept | -5.080581^***^ | -5.018082^***^ | -6.546128^***^ | -6.581111^***^ | -6.351259^***^ | -6.337110^***^ |
| lnsig2u | 0.456284 | 0.457856 | 0.328528 | 0.356545 | 0.386551 | 0.388225 |
| Observations | 2,388 | 2,388 | 2,388 | 2,388 | 2,388 | 2,388 |
| sigma_u | 1.256264 | 1.257251 | 1.178525 | 1.195151 | 1.213217 | 1.214233 |
| rho | 0.324194 | 0.324539 | 0.296855 | 0.302736 | 0.309107 | 0.309465 |
| p | 0.000000 | 0.000000 | 0.000000 | 0.000000 | 0.000000 | 0.000000 |
| bic | 6.92e+02 | 6.82e+02 | 6.73e+02 | 6.78e+02 | 6.85e+02 | 6.93e+02 |

^*^ *p* < 0.05, ^**^ *p* < 0.01, ^***^ *p* < 0.001; (U) = university level variable; ExIn = “excellence initiative”

Tab. 9b: Two-level logistic regression, second “initiative” phase (2012-2017), all universities

| DV=ExIn-funded | | | | | | | |
| --- | --- | --- | --- | --- | --- | --- | --- |
|  | Model 1 | Model 2 | Model 3 | Model 4 | Model 5 | Model 6 | Model 7 |
| Professors | 0.134104^***^ | 0.098283^***^ | 0.093758^***^ | 0.167302^***^ | 0.171687^***^ | 0.173286^***^ | 0.174681^***^ |
| Total grant funding |  | 0.119472^***^ | 0.111851^***^ | 0.124381^***^ | 0.070207^*^ | 0.068659 | 0.071790^*^ |
| DFG grant funding |  |  | 0.031678^**^ | 0.032247^**^ | 0.004028 | 0.012131 | 0.011722 |
| Students |  |  |  | -1.394023^***^ | -1.541516^***^ | -1.529333^***^ | -1.554121^***^ |
| Phase 1 |  |  |  |  | 6.040811^***^ | 6.048217^***^ | 6.073146^***^ |
| Founded after 1945 (U) |  |  |  |  |  | 0.640197 | 0.638906 |
| East Germany (U) |  |  |  |  |  |  | -0.332986 |
| Intercept | -4.931936^***^ | -4.912985^***^ | -5.657228^***^ | -5.756519^***^ | -5.738849^***^ | -6.242998^***^ | -6.203378^***^ |
| lnsig2u | -0.029492 | 0.201887 | 0.108190 | 0.171891 | -0.506117 | -0.637020 | -0.506778 |
| Observations | 2,396 | 2,396 | 2,396 | 2,396 | 2,396 | 2,396 | 2,396 |
| sigma_u | 0.985362 | 1.106214 | 1.055585 | 1.089747 | 0.776422 | 0.727232 | 0.776166 |
| rho | 0.227877 | 0.271117 | 0.253003 | 0.265231 | 0.154862 | 0.138493 | 0.154776 |
| p | 0.000000 | 0.000000 | 0.000000 | 0.000000 | 0.000000 | 0.000000 | 0.000000 |
| bic | 7.60e+02 | 7.44e+02 | 7.43e+02 | 7.09e+02 | 4.04e+02 | 4.10e+02 | 4.17e+02 |

^*^ *p* < 0.05, ^**^ *p* < 0.01, ^***^ *p* < 0.001; (U) = university level variable; ExIn = “excellence initiative”

Tab. 9c: Two-level logistic regression, first “initiative” phase (2006-2011), 12 subject fields with good WoS coverage, all universities

| DV=ExIn-funded | | | | | | | |
| --- | --- | --- | --- | --- | --- | --- | --- |
|  | Model 1 | Model 2 | Model 3 | Model 4 | Model 5 | Model 6 | Model 7 |
| Citations | 0.566880^***^ | 0.494364^***^ | 0.492519^***^ | 0.445917^***^ | 0.500020^***^ | 0.495780^***^ | 0.505399^***^ |
| Professors |  | 0.062377^**^ | 0.030574 | 0.026949 | 0.001290 | -0.001131 | -0.003900 |
| Total grant funding |  |  | 0.085760^*^ | 0.066730 | 0.066973 | 0.068932 | 0.069469 |
| DFG grant funding |  |  |  | 0.090206^**^ | 0.089567^**^ | 0.070416^*^ | 0.074635^*^ |
| Students |  |  |  |  | 0.368885 | 0.372392 | 0.398133 |
| Founded after 1945 (U) |  |  |  |  |  | -0.960572 | -0.906028 |
| East Germany (U) |  |  |  |  |  |  | 0.468667 |
| Intercept | -3.802962^***^ | -4.767178^***^ | -4.516534^***^ | -5.837735^***^ | -5.826572^***^ | -5.154399^***^ | -5.343520^***^ |
| lnsig2u | 0.381569 | 0.380839 | 0.331702 | 0.211194 | 0.294432 | 0.282858 | 0.268067 |
| Observations | 551 | 551 | 551 | 551 | 551 | 551 | 551 |
| sigma_u | 1.210199 | 1.209757 | 1.180397 | 1.111374 | 1.158604 | 1.151919 | 1.143431 |
| rho | 0.308044 | 0.307889 | 0.297518 | 0.272961 | 0.289788 | 0.287412 | 0.284392 |
| p | 0.000000 | 0.000000 | 0.000000 | 0.000000 | 0.000000 | 0.000000 | 0.000000 |
| bic | 2.97e+02 | 2.93e+02 | 2.93e+02 | 2.90e+02 | 2.95e+02 | 2.99e+02 | 3.04e+02 |

^*^ *p* < 0.05, ^**^ *p* < 0.01, ^***^ *p* < 0.001; (U) = university level variable; ExIn = “excellence initiative”

Tab. 9d: Two-level logistic regression, second “initiative” phase (2012-2017), 12 subject fields with good WoS coverage, all universities

| DV=ExIn-funded | | | | | | | | |
| --- | --- | --- | --- | --- | --- | --- | --- | --- |
|  | Model 1 | Model 2 | Model 3 | Model 4 | Model 5 | Model 6 | Model 7 | Model 8 |
| Citations | 0.230810^***^ | 0.359803^***^ | 0.356780^***^ | 0.345003^***^ | 0.290047^***^ | 0.219398^**^ | 0.227068^***^ | 0.230810^***^ |
| Professors | 0.120391^**^ | 0.081682^***^ | 0.058463^**^ | 0.054343^*^ | 0.096495^**^ | 0.118220^**^ | 0.122727^**^ | 0.120391^**^ |
| Total grant funding | 0.043532 |  | 0.057053^*^ | 0.054288^*^ | 0.082443^**^ | 0.048879 | 0.045786 | 0.043532 |
| DFG grant funding | -0.001245 |  |  | 0.016387 | 0.015873 | -0.010556 | -0.001592 | -0.001245 |
| Students | -0.865635 |  |  |  | -0.692342 | -0.919807 | -0.901012 | -0.865635 |
| Phase 1 | 4.665111^***^ |  |  |  |  | 4.638095^***^ | 4.688972^***^ | 4.665111^***^ |
| Founded after 1945 (U) | 0.860737 |  |  |  |  |  | 0.854493 | 0.860737 |
| East Germany (U) | 0.207269 |  |  |  |  |  |  | 0.207269 |
| Intercept | -6.008904^***^ | -4.946507^***^ | -4.873281^***^ | -5.189184^***^ | -5.296454^***^ | -5.283296^***^ | -5.980394^***^ | -6.008904^***^ |
| lnsig2u | -0.234567 | 0.062066 | 0.236544 | 0.243190 | 0.151644 | 0.008159 | -0.164644 | -0.234567 |
| Observations | 550 | 550 | 550 | 550 | 550 | 550 | 550 | 550 |
| sigma_u | 0.889333 | 1.031520 | 1.125550 | 1.129296 | 1.078771 | 1.004088 | 0.920975 | 0.889333 |
| rho | 0.193814 | 0.244386 | 0.278020 | 0.279356 | 0.261304 | 0.234569 | 0.204974 | 0.193814 |
| p | 0.000000 | 0.000000 | 0.000000 | 0.000000 | 0.000000 | 0.000000 | 0.000000 | 0.000000 |
| bic | 2.45e+02 | 3.05e+02 | 3.06e+02 | 3.11e+02 | 3.13e+02 | 2.34e+02 | 2.39e+02 | 2.45e+02 |

^*^ *p* < 0.05, ^**^ *p* < 0.01, ^***^ *p* < 0.001; (U) = university level variable; ExIn = “excellence initiative”
