# Supplementary material for: Field size as a predictor of “excellence.” The selection of subject fields in Germany’s Excellence Initiative
Source: PLoS One. 2025 Mar 11;20(3):e0300828. doi: 10.1371/journal.pone.0300828 (PMC11896035; doi:10.1371/journal.pone.0300828)
Supplement: S10 Appendix — (DOCX) [file pone.0300828.s010.docx]

# Appendix 10: Goodness of fit test

Tab. 10a: Goodness of fit test 2006-2011 (Hosmer-Lemeshow)

| Group | Prob | Obs_1 | Exp_1 | Obs_0 | Exp_0 | Total |
| --- | --- | --- | --- | --- | --- | --- |
| 1 | 0.008 | 0 | 1.7 | 239 | 237.3 | 239 |
| 2 | 0.010 | 0 | 2.1 | 239 | 236.9 | 239 |
| 3 | 0.013 | 2 | 2.7 | 237 | 236.3 | 239 |
| 4 | 0.016 | 0 | 3.4 | 239 | 235.6 | 239 |
| 5 | 0.020 | 5 | 4.3 | 233 | 233.7 | 238 |
| 6 | 0.026 | 6 | 5.4 | 233 | 233.6 | 239 |
| 7 | 0.034 | 4 | 7.1 | 235 | 231.9 | 239 |
| 8 | 0.048 | 11 | 9.7 | 228 | 229.3 | 239 |
| 9 | 0.084 | 22 | 14.6 | 217 | 224.4 | 239 |
| 10 | 0.996 | 52 | 51.1 | 186 | 186.9 | 238 |

Number of observations = 2,388
Number of groups = 10
Hosmer-Lemeshow chi2(8) = 13.14
Prob > chi2 = 0.1071

Tab. 10b: Goodness of fit test 2012-2017 (Hosmer-Lemeshow)

| Group | Prob | Obs_1 | Exp_1 | Obs_0 | Exp_0 | Total |
| --- | --- | --- | --- | --- | --- | --- |
| 1 | 0.004 | 0 | .6 | 240 | 239.4 | 240 |
| 2 | 0.005 | 0 | 1.1 | 240 | 238.9 | 240 |
| 3 | 0.005 | 1 | 1.2 | 239 | 238.8 | 240 |
| 4 | 0.006 | 1 | 1.3 | 238 | 237.7 | 239 |
| 5 | 0.006 | 0 | 1.5 | 239 | 237.5 | 239 |
| 6 | 0.008 | 1 | 1.7 | 239 | 238.3 | 240 |
| 7 | 0.011 | 2 | 2.2 | 238 | 237.8 | 240 |
| 8 | 0.018 | 3 | 3.3 | 236 | 235.7 | 239 |
| 9 | 0.045 | 9 | 6.7 | 231 | 233.3 | 240 |
| 10 | 1.000 | 108 | 105.6 | 131 | 133.4 | 239 |

Number of observations = 2,396

Number of groups = 10
Hosmer-Lemeshow chi2(8) = 4.49
Prob > chi2 = 0.8100
