# Supplementary material for: Field size as a predictor of “excellence.” The selection of subject fields in Germany’s Excellence Initiative
Source: PLoS One. 2025 Mar 11;20(3):e0300828. doi: 10.1371/journal.pone.0300828 (PMC11896035; doi:10.1371/journal.pone.0300828)
Supplement: S11 Appendix — (DOCX) [file pone.0300828.s011.docx]

# Appendix 11: Robustness checks

Tab. 11a: Logistic regression, first “initiative” phase (2006–2011), all universities, incl. citations.

|  | DV = Excellence Initiative funded | | | |
| --- | --- | --- | --- | --- |
|  | Model 1 | Model 2 | Model 3 | Model 4 |
| Professors | 0.105099^***^ | 0.079204^***^ | 0.057576^***^ | 0.039516^*^ |
| Total grant funding |  | 0.130803^***^ | 0.069347^*^ | 0.073081^*^ |
| Citations |  |  | 0.385166^***^ | 0.421349^***^ |
| Students |  |  |  | 0.254079 |
| Intercept | -4.281834^***^ | -4.223346^***^ | -4.214667^***^ | -4.219761^***^ |
| Observations | 2388 | 2388 | 2388 | 2388 |
| r2_p | 0.152671 | 0.173839 | 0.238831 | 0.241808 |

^*^ *p* < 0.05, ^**^ *p* < 0.01, ^***^ *p* < 0.001

Tab. 11b: Logistic regression, second “initiative” phase (2012–2017), all universities, incl. citations.

|  | DV = Excellence Initiative funded | | | | |
| --- | --- | --- | --- | --- | --- |
|  | Model 1 | Model 2 | Model 3 | Model 4 | Model 5 |
| Professors | 0.127277^***^ | 0.100775^***^ | 0.077238^***^ | 0.116906^***^ | 0.124363^***^ |
| Total grant funding |  | 0.083858^***^ | 0.037940 | 0.057827^**^ | 0.014639 |
| Citations |  |  | 0.267022^***^ | 0.211486^***^ | 0.201762^***^ |
| Students |  |  |  | -0.653553^**^ | -0.916477^*^ |
| Phase 1 |  |  |  |  | 5.867898^***^ |
| Intercept | -4.410611^***^ | -4.309952^***^ | -4.312490^***^ | -4.348694^***^ | -5.298393^***^ |
| Observations | 2396 | 2396 | 2396 | 2396 | 2396 |
| r2_p | 0.216103 | 0.231675 | 0.301258 | 0.310636 | 0.651632 |

^*^ *p* < 0.05, ^**^ *p* < 0.01, ^***^ *p* < 0.001

Table 11c: Linear regression (OLS), first “initiative” phase (2006–2011), all universities.

|  | DV = Excellence Initiative funded | | | |
| --- | --- | --- | --- | --- |
|  | Model 1 | Model 2 | Model 3 | Model 4 |
| Professors | 0.007015*** | 0.003750*** | 0.003498*** | 0.004407*** |
| Total grant funding |  | 0.018186*** | 0.017172*** | 0.016851*** |
| DFG grant funding |  |  | 0.001848*** | 0.001927*** |
| Students |  |  |  | -0.015319 |
| Intercept | -0.006732 | 0.000158 | -0.023450** | -0.023693** |
| Observations | 2388 | 2388 | 2388 | 2388 |
| r2 | 0.080594 | 0.115459 | 0.122551 | 0.123908 |

^*^ *p* < 0.05, ^**^ *p* < 0.01, ^***^ *p* < 0.001

Table 11d: Linear regression (OLS), second “initiative” phase (2012–2017), all universities.

|  | DV = Excellence Initiative funded | | | | |
| --- | --- | --- | --- | --- | --- |
|  | Model 1 | Model 2 | Model 3 | Model 4 | Model 5 |
| Professors | 0.009754*** | 0.005870*** | 0.005555*** | 0.009578*** | 0.004305*** |
| Total grant funding |  | 0.014696*** | 0.014177*** | 0.015199*** | 0.003105** |
| DFG grant funding |  |  | 0.000930*** | 0.000990*** | 0.000103 |
| Students |  |  |  | -0.076638*** | -0.035345*** |
| Phase 1 |  |  |  |  | 0.817932*** |
| Intercept | -0.017865** | -0.008706 | -0.027558*** | -0.023567** | -0.003522 |
| Observations | 2396 | 2396 | 2396 | 2396 | 2396 |
| r2 | 0.131106 | 0.166689 | 0.171580 | 0.193979 | 0.651472 |

^*^ *p* < 0.05, ^**^ *p* < 0.01, ^***^ *p* < 0.001

Table 11e: Logistic regression (logged variables), first “initiative” phase (2006–2011), all universities.

|  | DV = Excellence Initiative funded | | | |
| --- | --- | --- | --- | --- |
|  | Model 1 | Model 2 | Model 3 | Model 4 |
| Professors (log) | 1.490341*** | 0.748523*** | 0.728248*** | 0.763470*** |
| Total grant funding (log) |  | 1.044470*** | 0.855301*** | 0.850540*** |
| DFG grant funding (log) |  |  | 1.158362*** | 1.166570*** |
| Students (log) |  |  |  | -0.116913 |
| Intercept | -6.415434*** | -5.503732*** | -8.569057*** | -8.612783*** |
| Observations | 2388 | 2388 | 2388 | 2388 |
| r2 | 0.179136 | 0.226346 | 0.264008 | 0.264137 |

^*^ *p* < 0.05, ^**^ *p* < 0.01, ^***^ *p* < 0.001

Table 11f: Logistic regression (logged variables), second “initiative” phase (2012–2017), all universities.

|  | DV = Excellence Initiative funded | | | | |
| --- | --- | --- | --- | --- | --- |
|  | Model 1 | Model 2 | Model 3 | Model 4 | Model 5 |
| Professors (log) | 1.836237*** | 0.940053*** | 0.854283*** | 1.260922*** | 1.046553*** |
| Total grant funding (log) |  | 0.987974*** | 0.893684*** | 0.922505*** | 0.856784*** |
| DFG grant funding (log) |  |  | 0.730727*** | 0.785490*** | 0.260115 |
| Students (log) |  |  |  | -1.346496*** | -1.558971* |
| Phase 1 |  |  |  |  | 5.603957*** |
| Intercept | -7.154628*** | -5.940160*** | -7.992893*** | -8.537490*** | -7.240547*** |
| Observations | 2396 | 2396 | 2396 | 2396 | 2396 |
| r2 | 0.235475 | 0.282015 | 0.303253 | 0.315775 | 0.642545 |

^*^ *p* < 0.05, ^**^ *p* < 0.01, ^***^ *p* < 0.001

Table 11g: Logistic regression with total grant funding divided by quartile groups, second “initiative” phase (2012–2017), all universities.

|  | DV = Excellence Initiative funded | | | | |
| --- | --- | --- | --- | --- | --- |
|  | Model 1 | Model 2 | Model 3 | Model 4 | Model 5 |
| Professors | 0.127277*** | 0.073207*** | 0.063840*** | 0.116289*** | 0.120890*** |
| Total grant funding (p=2) |  | -0.751509 | -0.688802 | -0.721323 | -0.592412 |
| Total grant funding (p=3) |  | -0.174152 | -0.149716 | -0.198011 | -0.233630 |
| Total grant funding (p=4) |  | 1.702883** | 1.671531** | 1.587986* | 1.213674 |
| Total grant funding (p=5) |  | 2.457565*** | 2.433874*** | 2.128084*** | 1.637612* |
| DFG grant funding |  |  | 0.020469*** | 0.020810*** | 0.003807 |
| Students |  |  |  | -0.752426*** | -1.031804*** |
| Phase 1 |  |  |  |  | 5.744027*** |
| Intercept | -4.410611*** | -5.343617*** | -5.804237*** | -5.790879*** | -5.948403*** |
| Observations | 2396 | 2396 | 2396 | 2396 | 2396 |
| r2 | 0.216103 | 0.267438 | 0.280417 | 0.301738 | 0.649292 |

^*^ *p* < 0.05, ^**^ *p* < 0.01, ^***^ *p* < 0.001
