# Supplementary material for: Field size as a predictor of “excellence.” The selection of subject fields in Germany’s Excellence Initiative
Source: PLoS One. 2025 Mar 11;20(3):e0300828. doi: 10.1371/journal.pone.0300828 (PMC11896035; doi:10.1371/journal.pone.0300828)
Supplement: S12 Appendix — (DOCX) [file pone.0300828.s012.docx]

# Appendix 12: Logistic regression analyses with full samples

Table 12a: Logistic regression, first “initiative” phase (2006–2011), full sample (n = 2,829), all universities.

|  | DV = Excellence Initiative funded | | | |
| --- | --- | --- | --- | --- |
|  | Model 1 | Model 2 | Model 3 | Model 4 |
| Professors | 0.112307*** | 0.087027*** | 0.082994*** | 0.095154*** |
| Total grant funding |  | 0.130324*** | 0.096640** | 0.088886** |
| DFG grant funding |  |  | 0.057533*** | 0.058294*** |
| Students |  |  |  | -0.192628 |
| Intercept | -4.453984*** | -4.406335*** | -5.312870*** | -5.321830*** |
| Observations | 2829 | 2829 | 2829 | 2829 |
| r2 | 0.174072 | 0.193710 | 0.220302 | 0.222288 |

^*^ *p* < 0.05, ^**^ *p* < 0.01, ^***^ *p* < 0.001

Table 12b: Logistic regression, second “initiative” phase (2012–2017), full sample (n = 2,829), all universities

|  | Model 1 | Model 2 | Model 3 | Model 4 | Model 5 |
| --- | --- | --- | --- | --- | --- |
| Professors | 0.134047*** | 0.108576*** | 0.100564*** | 0.166730*** | 0.175328*** |
| Total grant funding |  | 0.082210*** | 0.074808** | 0.088956*** | 0.052895 |
| DFG grant funding |  |  | 0.020595*** | 0.020055*** | 0.003794 |
| Students |  |  |  | -1.258470*** | -1.517179*** |
| Phase 1 |  |  |  |  | 5.950006*** |
| Intercept | -4.567623*** | -4.480084*** | -4.953418*** | -5.006185*** | -5.559139*** |
| Observations | 2829 | 2829 | 2829 | 2829 | 2829 |
| r2 | 0.239250 | 0.253252 | 0.265395 | 0.306916 | 0.651541 |

^*^ *p* < 0.05, ^**^ *p* < 0.01, ^***^ *p* < 0.001p
